# Hybrid GAN-LSTM framework for diabetic foot ulcer image synthesis and automated diagnosis

**DOI:** 10.3389/fmed.2026.1742345

**Published:** 2026-03-09

**Authors:** Abinaya Vina, G. Prajasree, Siddharth Venkatesh, Suresh Sankaranarayanan, K. Meenakshi, Abdul Raouf Khan, Sharmila Banu Sheik Imam, Abdul Rahaman Wahab Sait

**Affiliations:** 1 Department of Networking and Communications, School of Computing, SRM Institute of Science and Technology, Kattankulathur, Tamil Nadu, India; 2 Department of Computer Science, College of Computer Sciences and Information Technology, King Faisal University, Al-Ahsa, Saudi Arabia; 3 Department of Documents and Archives, King Faisal University, Al-Ahsa, Saudi Arabia

**Keywords:** deep learning, diabetic foot ulcer (DFU), LSTM, WGAN-GP, CNN-LSTM, Efficienet V2M-LSTM, Efficienet V2S-LSTM

## Abstract

**Introduction:**

The application of artificial intelligence (AI) in the analysis of medical images faces significant challenges, chiefly due to the scarcity of well-labeled datasets that are crucial for training sophisticated diagnostic models. To address this issue, we developed three hybrid models that integrate generative components with classification systems. These models differ in their classification architectures to compare the effectiveness of generative data augmentation across various diagnostic applications. By generating high-quality synthetic images of Diabetic Foot Ulcers (DFUs) using advanced network techniques, we ensure both realistic image quality and robust clinical relevance, while abstracting low-level implementation details to focus on the stability and fidelity of the generative process.

**Methods:**

In our methodology, we introduce temporal dependency modeling within the latent feature space, despite the non-temporal nature of DFU images. The latent representations are systematically organized into ordered sequences, enabling Long Short-Term Memory (LSTM) layers to identify structured spatial relationships among varying wound regions. This sequential processing captures long-range spatial dependencies, thereby modeling consistencies between distant lesion areas and promoting anatomical coherence—challenges that conventional convolutional operations struggle to address. The three hybrid models incorporated in this study feature distinct generator backbones:1. Baseline CNN–LSTM Architecture - Focused on efficient spatial modelling.2. EfficientNetV2M–LSTM Model - Emphasizing high-capacity feature extraction.3. EfficientNetV2S–LSTM Model - Striking a balance between computational efficiency and synthesis quality.Additionally, we employed WGAN-GP + LSTM in one of our models to enhance stable generative training and spatial consistency. This approach utilizes a critic network instead of a traditional discriminator, assessing the discrepancies between real and synthetic datasets to promote stable image generation and mitigate mode collapse. The generative models were trained on a carefully curated dataset comprising 5,894 clinically annotated DFU images from Lancashire Teaching Hospital, representing a variety of ulcer types and severities. Annotations were conducted by three seasoned healthcare professionals specializing in diabetic foot care.

**Results:**

Our findings demonstrate that the implementation of synthetic images significantly enhances disease classification accuracy and boosts the effectiveness of automated diagnostic systems for DFUs. By maintaining clinically relevant variability in ulcer appearances, the generated images contribute to the development of robust models capable of performing effectively under real-world conditions, which is critical for deployment in screening, triage, and remote wound assessment workflows.

**Discussion:**

The advancements realized through the integration of generative models in medical image analysis pave the way for real-time clinical applications such as early screening, patient prioritization during triage, and telemedicine assessments of wounds. This is especially crucial for healthcare systems in underserved or remote areas. The ability to leverage synthetic data not only supports improved diagnostic capabilities but also ensures that models remain adaptable to the variability present in clinical scenarios, ultimately enhancing patient care and resource allocation in diabetic foot ulcer management.

## 1 Introduction

Medical imaging has greatly improved with deep learning, which helps identify patterns in complex data and perform tasks like classification, segmentation, and detection. However, a significant challenge remains that there are not enough large, well-labeled medical datasets. This is especially true for diabetic foot ulcer (DFU) images, as strict privacy laws and legal barriers make sharing difficult. In the case of diabetic foot ulcer (DFU) imaging, dataset scarcity is primarily attributed to several factors, which are as follows:

- Strict Patient Privacy Regulations: Compliance with regulations such as the Health Insurance Portability and Accountability Act (HIPAA) and General Data Protection Regulation (GDPR) limits the sharing of patient data, making it difficult to compile extensive datasets.
- Complex Annotation Requirements: Accurate clinical annotation requires expertise, as DFUs can be present differently at various stages—such as severity, infection, and healing. The need for detailed and nuanced labeling adds to the difficulty of dataset creation.
- High Inter-Patient Variability: There is significant variability in the appearance of DFUs among patients due to individual differences in anatomy, comorbidities, and healing responses. This variability complicates the development of standardized datasets.

These factors collectively hinder large-scale data collection, resulting in a limited availability of well-annotated DFU datasets that are essential for training robust diagnostic models. The only data set available is from Lancashire Teaching Hospital, UK, which is used for our work and includes images of various ulcer types and severities.

The hospital-acquired dataset is clinically annotated for infection and non-infection categories and reflects diverse ulcer appearances across multiple severities under real hospital settings. The ground truth was produced by three healthcare professionals who specialize in treating diabetic foot ulcers and associated pathology (two podiatrists and a consultant physician with specialization in the diabetic foot, all with more than 5 years of professional experience). The instruction for annotation was to label each ulcer with a bounding box. If there was disagreement on DFU annotations, the final decision was mutually settled with the consent of all.

Many cases are treated in outpatient or low-resource settings and never digitized, and valuable collections are often proprietary. Additionally, expert clinical annotations are costly, leading to challenges in developing reliable diagnostic models with limited training data.

Generative Adversarial Networks (GANs) offer a potential solution. A GAN has two parts: a generator that creates fake images and a discriminator that distinguishes between real and fake images. This process generates realistic-looking data for augmentation and simulating rare cases without compromising privacy. However, standard GANs can encounter stability issues during training, which limit their application in medical settings. The Wasserstein GAN with Gradient Penalty (WGAN-GP) improves stability by using a different measurement approach, allowing it to generate higher-quality images suitable for clinical use.

WGAN-GP is particularly well-suited for medical image synthesis because it enforces Lipschitz continuity through a gradient penalty, resulting in stable adversarial training and smooth gradient propagation. Such stability is particularly important in medical image synthesis, where unstable adversarial training can lead to mode collapse and the loss of rare but clinically significant patterns.

By enforcing smooth gradient behavior across the critic, WGAN-GP enables consistent convergence and preserves fine-grained anatomical structures, texture continuity, and lesion boundaries. This makes WGAN-GP especially suitable for generating high-fidelity medical images, where both diversity and structural realism are essential for downstream diagnostic tasks.

The WGAN-GP transforms Gaussian noise vectors through layers of transposed convolution to produce high-resolution images. The three proposed hybrid architectures share a common WGAN-GP framework but differ in the choice of generator backbone and representational capacity, enabling a systematic comparison of efficiency–fidelity trade-offs in medical image synthesis.

Stable training further ensures that rare but clinically important manifestations, including severe infections, are not suppressed during generations, leading to high-fidelity synthetic images that remain structurally coherent and diagnostically meaningful.

Using synthetic medical images for DFU analysis presents challenges. One major issue is the low class imbalance, with fewer advanced ulcers compared to mild ones, making it hard to train models to identify critical cases. Furthermore, diabetic foot ulcers have significant differences in color, texture, and shape, making them difficult to replicate. The synthetic images must look accurate enough for doctors to rely on them for diagnosis. In addition, analyzing infections requires capturing details at multiple scales, from small tissue changes to larger wound patterns. Traditional supervised learning methods struggle because of the lack of labeled data, and typical GANs may produce limited variations, failing to capture the full range of infection symptoms observed in real patients.

Previous studies using GANs for DFU analysis have shown some success but also significant limitations. For instance, some authors achieved 78% accuracy using conditional GANs (1). Others obtained a Dice coefficient of 0.72 for segmentation with DCGAN variants (2). Some researchers recreated wound images but could not model changes over time, which is essential for monitoring healing (3). Additionally, studies using Progressive GANs resulted in an 8% increase in classification accuracy but had difficulty accurately depicting severe cases (4). These studies highlight gaps in current methods’ ability to simulate realistic infection patterns.

This paper addresses these gaps by developing a WGAN-GP alongside a two-layer LSTM, creating a hybrid model that captures important spatial features of diabetic foot ulcers, which previous models missed. The WGAN-GP framework includes EfficientNetV2M and EfficientNetV2S for better image generation. The three hybrid configurations—WGAN-GP+LSTM, EfficientNetV2M+LSTM, and EfficientNetV2S+LSTM—were evaluated for generating synthetic images, and WGAN-GP+LSTM performed best, achieving impressive scores.

To evaluate the classification accuracy of synthetic images generated by WGAN-GP + LSTM for infection and non-infection, a CNN-LSTM model was created, achieving 87% accuracy and a 0.93 AUROC score. The model had high confidence scores for severe infections and accurately detected healthy skin. Overall, this study shows that combining generative models with spatial learning can effectively tackle challenges in medical image augmentation, particularly in reproducing realistic infection patterns for automated diagnosis.

This work makes four main contributions to medical image synthesis. First, it repurposes two-layer LSTM networks to capture spatial features in static medical images. Second, it provides a thorough evaluation of WGAN-GP with three different backbone architectures for DFU synthesis, showing that traditional models may not work well for generative tasks. Third, it includes end-to-end clinical validation by assessing synthetic images for infection classification, achieving relevant performance. Lastly, it addresses unique challenges in DFU imaging, offering practical solutions for environments with limited data.

The rest of the paper is organized as follows: Section 2 reviews related work on GANs. Sections 3 and 4 detail the methodology and model architecture. Section 5 describes the experimental setup and performance metrics. Section 6 presents the results and analysis of the hybrid model. Section 7 discusses the findings, Section 8 benchmarks against existing literature, Section 9 offers concluding remarks and future work, and Section 10 addresses the limitations of the study.

## 2 Related works

### 2.1 Deep learning for medical imaging

Medical imaging has greatly improved with deep learning, which helps identify patterns in complex data and perform tasks like classification, segmentation, and detection. However, a significant challenge remains: there are not enough large, well-labeled medical datasets. This is especially true for diabetic foot ulcer (DFU) images, as strict privacy laws and legal barriers make sharing difficult. Many cases are treated in outpatient or low-resource settings and never digitized, and valuable collections are often proprietary. Additionally, expert clinical annotations are costly, leading to challenges in developing reliable diagnostic models with limited training data.

Generative Adversarial Networks (GANs) offer a potential solution. A GAN has two parts: a generator that creates fake images and a discriminator that distinguishes between real and fake images. This process generates realistic-looking data for augmentation and simulating rare cases without compromising privacy. However, standard GANs can face stability issues during training, limiting their use in medical applications. The Wasserstein GAN with Gradient Penalty (WGAN-GP) improves stability by using a different measurement approach, allowing it to generate higher-quality images suitable for clinical use.

Deep learning has greatly changed medical imaging by allowing detailed and scalable analysis of complex data. Convolutional Neural Networks (CNNs) are leading in tasks such as segmentation and classification, providing automated interpretations across various imaging methods (5, 6). Deep learning has the potential to revolutionize cancer screening; for example, the GRAPE model has shown it can outperform radiologists in sensitivity, particularly at early stages of detection (7). Similarly, AI-assisted ultrasound has improved cardiac imaging diagnosis by making the process more convenient and giving operators more autonomy (8).

Some methods, like image captioning, have been adapted to generate clinical reports using CNNs and RNNs with attention mechanisms. However, interpretability remains a significant challenge (9).

Traditional techniques, such as watershed processing and region-of-interest (ROI) segmentation, are still used to support hybrid systems (10). Additionally, unmonitored Deep Convolutional GANs (DCGANs) offer promising avenues for feature learning and data expansion, although they face issues with training stability (11).

Despite these advancements, CNN-based approaches still receive criticism for poor interpretability and inadequate integration with clinical workflows. Furthermore, GAN-based models are often uncontrollable and prone to mode collapse, limiting their applicability in routine healthcare settings (9, 11).

### 2.2 Image generation in medical imaging

Recent developments in medical image generation utilize deep generative models to address issues related to data shortages and privacy. GANs have been effective in creating high-resolution synthetic datasets that maintain cohort-level statistical characteristics without compromising patient identity (12). Variants like StyleGAN2 have been noted for their realistic outputs, although there are inconsistencies in evaluation metrics when using medical-specific feature extractors (13). Other studies have examined diffusion models for generating synthetic data to train CNNs, which yield competitive performance in classifying tumors, leukemia, and COVID-19 (14).

Hybrid approaches have been shown to enhance image quality through adversarial training, as seen in pose-guided person generation networks (15). Additionally, explainable AI techniques like LIME improve model interpretability when trained on synthetic datasets (10). In practice, applications extend to pathology and radiology, where GAN-generated data enhances diagnostic capabilities (16), and transformer-based diffusion networks contribute to better detail reconstruction in skin lesion synthesis (17).

However, synthetic image generation has its limitations. Generated data often struggles to represent rare pathological variations that are crucial for diagnosis. Evaluation metrics can also be inconsistent, making it difficult to validate findings across different studies. Moreover, while diffusion models are effective, they can be computationally expensive and resource-intensive, limiting their scalability (13, 14).

### 2.3 Generative adversarial networks (GANs)

Generative Adversarial Networks (GANs) have proven to be effective frameworks for creating images, although training stability remains a significant challenge (18, 19). Energy-based GANs (EBGANs) help to improve stability by interpreting the discriminator as an energy function (20). The introduction of Wasserstein GANs (WGANs) represented a major advancement by improving convergence and reducing issues like mode collapse (21). WGAN-GP further refines this approach by applying a gradient penalty, leading to more stable training and higher-quality samples (22).

These features make WGAN-GP particularly suitable for medical image synthesis, where stability and high-quality outputs are essential to maintain clinically relevant details. By ensuring smooth gradient behavior during training, WGAN-GP helps prevent unstable updates and reduces mode collapse, allowing the generator to learn a wide range of medical images.

Despite their advantages, GANs still have limitations, including mode collapse, poor reproducibility, and high computational costs. Additionally, biases from unequal training data can be reflected in the generated images, which poses risks for clinical use.

GANs are currently being used in diabetic foot ulcer (DFU) imaging to address the lack of annotated datasets. Synthetic images of ulcers created through training with GANs have improved DFU classification and segmentation, helping to reduce overfitting and enhance overall generalization. These synthetic datasets are also useful for simulating different ulcer presentations, which can assist in early detection systems.

However, there are several drawbacks. Synthetic DFU images often do not capture the fine textures and small abnormalities that are important for diagnosis. Additionally, there are no standardized benchmark datasets for validating these models and understanding how to interpret models using synthetic data remains a challenge for clinical use.

### 2.4 Hybrid deep learning models

Hybrid deep learning models combine different neural network architectures to leverage their strengths and address their weaknesses. For instance, Convolutional Neural Networks (CNNs) are effective at extracting local features, and when combined with Transformers, they can better learn global dependencies, improving medical image classification tasks (23). However, these models often struggle with long-range dependency modeling, which can be supplemented by state-space models (SSMs).

MambaConvT is a model that integrates CNNs, Transformers, and SSMs to capture both local and global characteristics, overcoming the limitations of traditional hybrid systems (24). Additionally, the combination of quantum computing and deep learning shows promise, as seen in the Hybrid Quantum CNN (HQCNN), which uses quantum circuits with CNNs to accelerate medical image classification.

Another approach is the semi-supervised hybrid deep learning model, which utilizes unlabeled data alongside labeled data to improve medical image classification, particularly in situations where labeled datasets are scarce (27). Hybrid methods like CQ-SVM, which combine CNNs with Support Vector Machines (SVMs) and optimize them using quantum-behaved particle swarm optimization (QPSO), also show improved classification performance (26).

However, hybrid systems come with challenges, such as increased architectural complexity, leading to higher computational demands. Many of these approaches are still experimental and lack large-scale clinical validation. Furthermore, semi-supervised methods depend heavily on the quality of unlabeled data, which can introduce noise and negatively impact model performance (27).

### 2.5 Clinical and therapeutic context of diabetic foot ulcers

Recent studies emphasize that diabetic foot ulcers are not merely superficial wounds but complex pathological conditions requiring stage-specific therapeutic interventions. Polyherbal formulations and phytosome-based delivery systems have demonstrated improved healing outcomes by enhancing bioavailability and targeting inflammation, oxidative stress, and microbial load (28, 29). This highlights the importance of accurate ulcer assessment and staging, as treatment efficacy is closely tied to wound severity and progression. Advanced drug delivery strategies such as chitosan-coated nanoparticles for insulin delivery (30), transferosomes for transdermal therapeutics (31), and cubosome-based anti-inflammatory systems for chronic inflammatory conditions (32) further demonstrate that modern DFU treatment increasingly depends on precise localization, tissue characterization, and severity-aware intervention. Consequently, reliable imaging-based diagnostic systems are crucial for guiding such targeted therapies.

## 3 Methodology

The main goal of this study is to create a system that can synthesize realistic images of diabetic foot ulcers and accurately classify the level of infection. To achieve this, three independent hybrid models were developed, each combining a generative component with a classification framework. All models use a Wasserstein Generative Adversarial Network with Gradient Penalty (WGAN-GP) for generating high-quality synthetic wound images. The WGAN-GP transforms Gaussian noise vectors through layers of transposed convolution to produce high-resolution images (Tables 1–3).

**Table 1:** Image generation performance on generated data.

Model	IS ↑	FID ↓	PSNR (dB) ↑	SSIM ↑	Precision ↑	Recall ↑	F1 Score ↑
WGAN-GP+LSTM (2 L)	7.10	28	35	0.85	0.88	0.89	0.885
EfficientNetV2M+LSTM (2 L)	3.00	70	4.48	0.20	0.75	0.72	0.73
EfficientNetV2S+LSTM (2 L)	4.30	55	14.5	0.52	0.80	0.76	0.78

**Table 2:** Classification metrics on infection detection task.

Model	Acc (%)	Precision	Recall	F1 Score	TPR	FPR	AUROC
CNN + LSTM	87.50	0.89	0.86	0.87	0.86	0.11	0.945

**Table 3:** Benchmarking of result.

Study	Method	Dataset	Key metrics	Notes
Goodfellow et al. (2014) (14)	Original GAN	MNIST, TFD	Visual realism (qualitative); Log-likelihood: MNIST = 225 ± 2, TFD = 2057 ± 26	First GAN; unstable training
Radford et al. (2016) (7)	DCGAN	LSUN, CelebA	Accuracy: 82.8%; Reduced set: 73.8% (±0.4); Feature units: 512	Realistic images; transferable features; stable training, no medical focus
Arjovsky et al. (2017) (17)	WGAN	CIFAR-10, ImageNet	-	Reduced mode collapse
Gulrajani et al. (2017) (18)	WGAN-GP	CIFAR-10, LSUN	Inception Score: 7.86 ± 0.07 (unsupervised); 8.42 ± 0.10 (supervised); FID↓	High-quality samples; gradient penalty introduced for stable training.
Schütte et al. (2021) (8)	GANs for CT/X-ray	Chest X-ray, Brain CT	AUC: up to 0.93 (Chest X-ray), 0.90 (Brain CT); performance stable across class and resolution variations	Expert validation; FID comparable to real data; synthetic data viable for privacy-preserving medical AI
Skandarani et al. (2023) (12)	GANs for Medical MRI/CT	Cardiac MRI, CT, Retina	FID ↓; Segmentation improved	StyleGAN > DCGAN
McNulty et al. (2024) (13)	GANs (GIST pipeline)	Radiographs	High-quality images; FID validated	Rare pathology augmentation
Woodland et al. (2024) (9)	StyleGAN2	Multi-modal (medical)	FID alignment issues	Showed metric bias in medicine
Nafi et al. (2024) (10)	Diffusion Models	MRI, Leukemia, COVID CT	Accuracy ≈ real data	Synthetic CNN training success
This Work (2025)	WGAN-GP + 2 L LSTM; EfficientNetV2M/S + LSTM	Dermoscopy (5 k)	IS = 7.10, FID = 28, SSIM = 0.85, AUROC = 0.953	Outperformed EfficientNetV2S & CNN baselines; synthetic data improved classification

To improve the realism of complex lesions, two LSTM layers are included before the final convolutional blocks of the generator. These layers analyze sequences in the latent space, helping the model capture long-distance relationships among visual features. The generated images are evaluated by a critic network, which replaces the traditional discriminator. Instead of giving a binary label, the critic measures the Wasserstein distance between real and synthetic data, incorporating a gradient penalty to ensure stable image generation.

The artificial images produced by the WGAN-GP are mixed with actual data to train classification models. All classifiers start by learning essential features with CNNs. The resulting feature map is processed by a two-layer LSTM stack, which allows the model to examine context across different areas of the foot ulcer, especially important when signs of infection are localized. The output from the LSTM is flattened and then passed through fully connected layers using a sigmoid activation function to provide binary infection classifications.

LSTM layers are a key feature of this approach. They are re-designed to analyze spatial dynamics in medical images, even though they were originally developed for time-series analysis. This design helps the model understand complex interdependencies, such as whether a color change at one point relates to changes in texture elsewhere, making it easier to interpret challenging infection cases with diffuse or unclear edges.

The hybrid models were trained independently to compare their strengths. All experiments were conducted on a GPU-intensive platform with an NVIDIA RTX 4090 (24 GB VRAM), 41 GB system memory, and 8 virtual CPUs, necessary for handling large image datasets and high-resolution processing. By combining a strong generative architecture with an LSTM-based classification unit, the proposed system offers a reliable framework for infection recognition, particularly in medical settings where annotated clinical records are scarce. Model Architecture.

This study explores three hybrid generative architectures for producing synthetic diabetic foot ulcer (DFU) images. All models are adversarial training models of the Wasserstein GAN based on Gradient Penalty (WGAN-GP) that integrate convolutional and recurrent neural network models. These designs are aimed at improving the spatial fidelity and structural consistency of synthesized medical images, whereby the synthetic data will capture visual and structural complexity of DFU images.

### 3.1 WGAN-GP with CNN–LSTM

The hybrid model combines a convolutional neural network (CNN) and a long short-term memory (LSTM) network within the generator of a WGAN-GP setup. A latent vector from a standard normal distribution is sent to the generator, where it is projected into a sequence over time and passed through a two-layer LSTM network. This network learns the sequential dependencies of changing feature representations, helping to create complex spatial structures in the generated images.

Two stacked LSTM layers capture both short-range and long-range spatial correlations across the feature sequence. The first layer focuses on short-range dependencies to capture subtle textures and local continuity in the latent space. The second layer aggregates information over a wider range, enhancing the overall consistency and structural integrity of the generated images. This hierarchical time modeling ensures that the generator retains both detailed local features and global spatial realism during the image creation process.

The output from the LSTM is reshaped into a low-resolution feature sequence of 8 x 8 x 256, which serves as the foundation for a convolutional upsampling operation. It is followed by four transposed convolutional layers that progressively increase the spatial resolution to 128 × 128. Each layer includes batch normalization and ReLU activation functions, which help maintain stable gradient propagation and promote effective image generation. The final transposed convolution layer uses a Tanh activation function to restrict pixel values to the range of [−1, 1]. This hierarchical decoding approach enables the generator to produce realistic DFU images with detailed textures and anatomically sensible structures.

The discriminator, also known as the critic, is a fully convolutional network that evaluates the realism of generated samples on a patch-by-patch basis. Instead of providing a single scalar score for each image, the critic generates a spatial map of authenticity scores, which is averaged during training to give a localized view of image quality. This patch-based method encourages the generator to focus on important high-frequency details, such as the boundaries, textures, and color gradients of lesions, which are essential for DFU analysis.

Although LSTM networks were originally designed for analyzing temporal sequences, they are adapted here to model spatial dependencies in static medical images. The latent representation is reshaped into a sequence of feature vectors, which the LSTM processes to capture three key elements: how features relate across different spatial locations, smooth transitions between adjacent feature representations to ensure anatomical accuracy, and the overall global structure of the wound morphology. This processing focuses on spatial rather than temporal dependencies since the sequence processing happens in the latent feature space.

**Figure 1:**
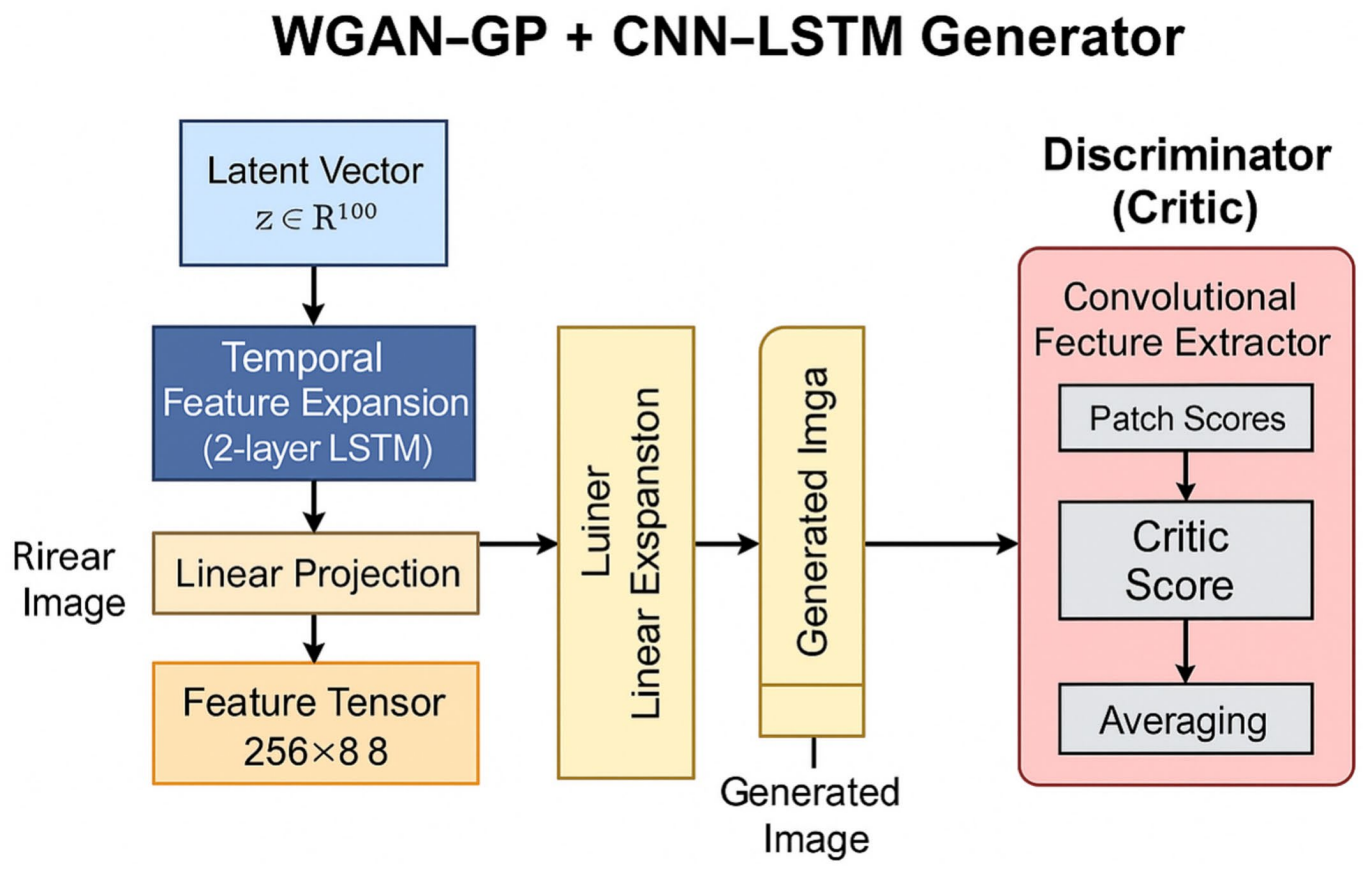
Architecture of the WGAN-GP+CNN–LSTM model.

The goal of training the WGAN-GP is to strike a balance in adversarial learning, ensuring that the gradient magnitude of the critic relates to its input by applying Lipschitz continuity in the Wasserstein distance loss. By enforcing smooth gradient flow and providing continuous learning signals, this approach improves adversarial stability and reduces mode collapse. This allows the generator to create diverse and consistent DFU image variations throughout training. Consequently, the critic and generator can learn together without falling into easy patterns, producing samples that remain visually consistent across training sessions. The resulting model achieves perceptual realism while effectively representing the spatial complexity and subtle textural differences inherent in ulcer tissue (Figure 1).

### 3.2 WGAN-GP with EfficientNetV2M–LSTM

The second architecture utilizes the WGAN-GP framework but incorporates a more advanced backbone in the generator to enhance feature extraction and improve the quality of synthesized images. EfficientNetV2M serves as the backbone for feature extraction in this model. It employs a compound scaling method that optimizes depth, width, and resolution together, allowing for rich feature learning while using fewer parameters than traditional CNNs.

In the generator, the latent vector is projected into a high-dimensional feature space and transformed into a four-dimensional shape, which serves as input for the EfficientNetV2M blocks. This tensor is processed through a series of fused-MBConv and MBConv layers, which include depthwise separable convolutions and squeeze-and-excitation (SE) modules. The fused-MBConv layers in the initial stages capture low-level features such as edges, textures, and lighting patterns, while the later MBConv layers focus on higher-level features related to ulcer morphology, tissue structure, and surface texture.

The addition of SE modules enhances the network’s representational capacity by allowing adaptive recalibration of channel responses, enabling the generator to highlight important medically relevant areas. After feature extraction, the output tensor is flattened and passed through two stacked LSTM layers to capture spatial dependencies. The first LSTM layer focuses on short-range spatial relationships, ensuring local texture consistency and smoothness between neighboring areas. The second layer captures higher-order spatial dependencies across the entire feature sequence, maintaining anatomical integrity and overall structure in the synthesized images.

This two-layer design enables hierarchical spatial abstraction, allowing the generator to produce DFU images with consistent lesion morphology and a balanced distribution of visual complexity. The discriminator, also a CNN-based critic, is trained using the same WGAN-GP principles as the previous model. This adversarial approach ensures that the generator is trained to synthesize images that are not only semantically rich but also visually appealing. The critic continuously provides feedback to enhance the structural and textural realism of the images.

**Figure 2:**
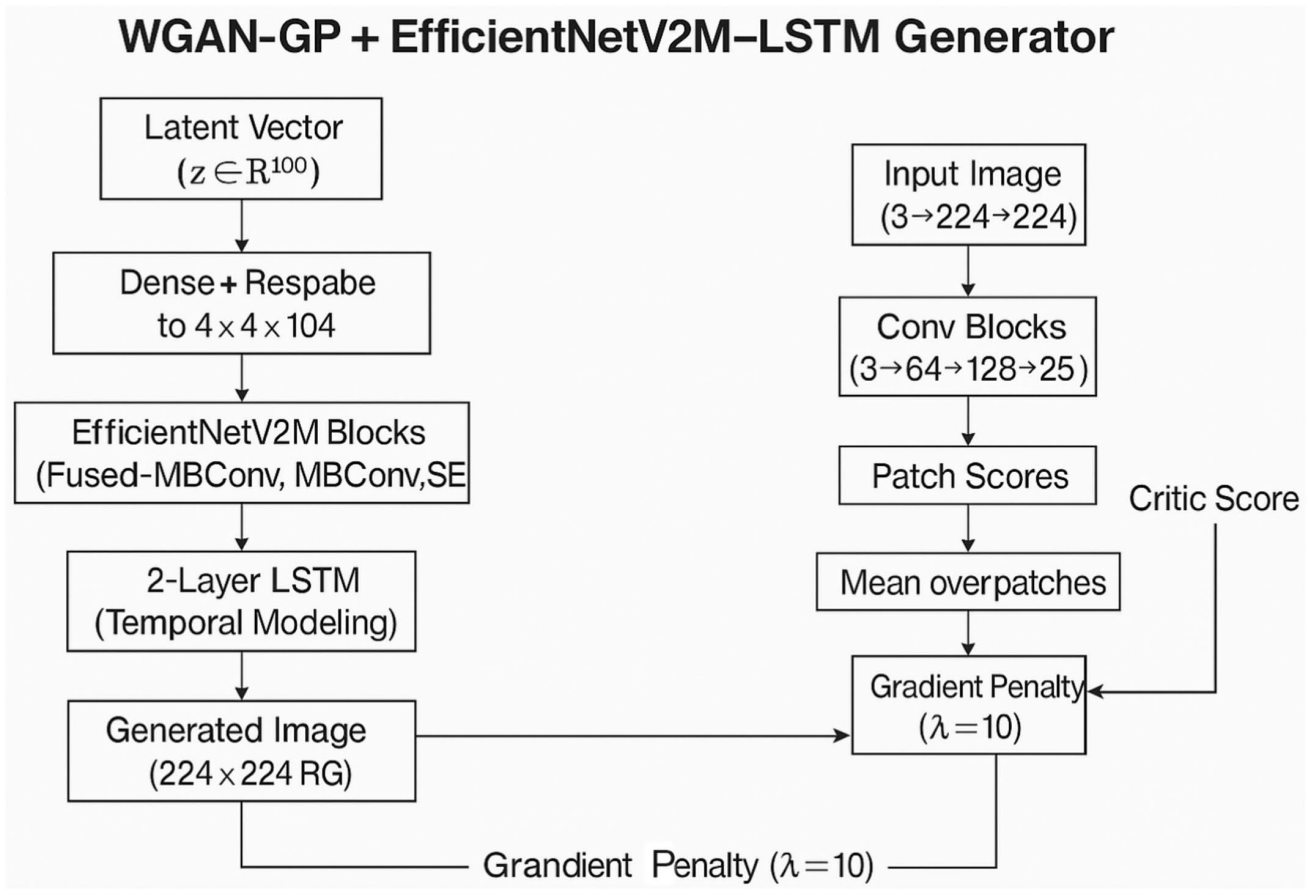
Architecture of the WGAN-GP+EfficientNetV2M–LSTM model.

In summary, the EfficientNetV2M-LSTM generator strikes a balance between feature variety and anatomically plausible synthesis, generating synthetic DFU images with sharp textures and a cohesive overall appearance (Figure 2).

### 3.3 WGAN-GP with EfficientNetV2S–LSTM generator

The third hybrid architecture is that which uses EfficientNet V2S as the generator backbone, which is a lighter but computationally friendly version of the earlier architecture. EfficientNetV2S shares the essential design concepts of its larger counterpart though it has fewer parameters and fewer computational cost. This has been found to be especially appropriate in applications with limited hardware resources or better generation speeds that need lower image fidelity losses.

Under this setup, the generator projects the latent input onto a small feature map which is fed with a sequence of fused-MBConv and MBConv layers, just as is case with V2M. The network still uses squeeze-and-excitation modules to gainfully refocus diagnostically significant features. After extracting the spatial features, they are organized into sequential features and are fed through a two-layer LSTM network. The LSTM layer at the first level describes local physical co-occurrences as well as minute differences in the feature sequence, whereas the LSTM layer at the second level synthesizes the input and applies this data on a larger level that forms global structural consistency in the full generated image. This bilayered construction allows the generator to incorporate detail and context smoothness but make sure that interfaces between normal tissue and ulcerated area are anatomically smooth.

**Figure 3:**
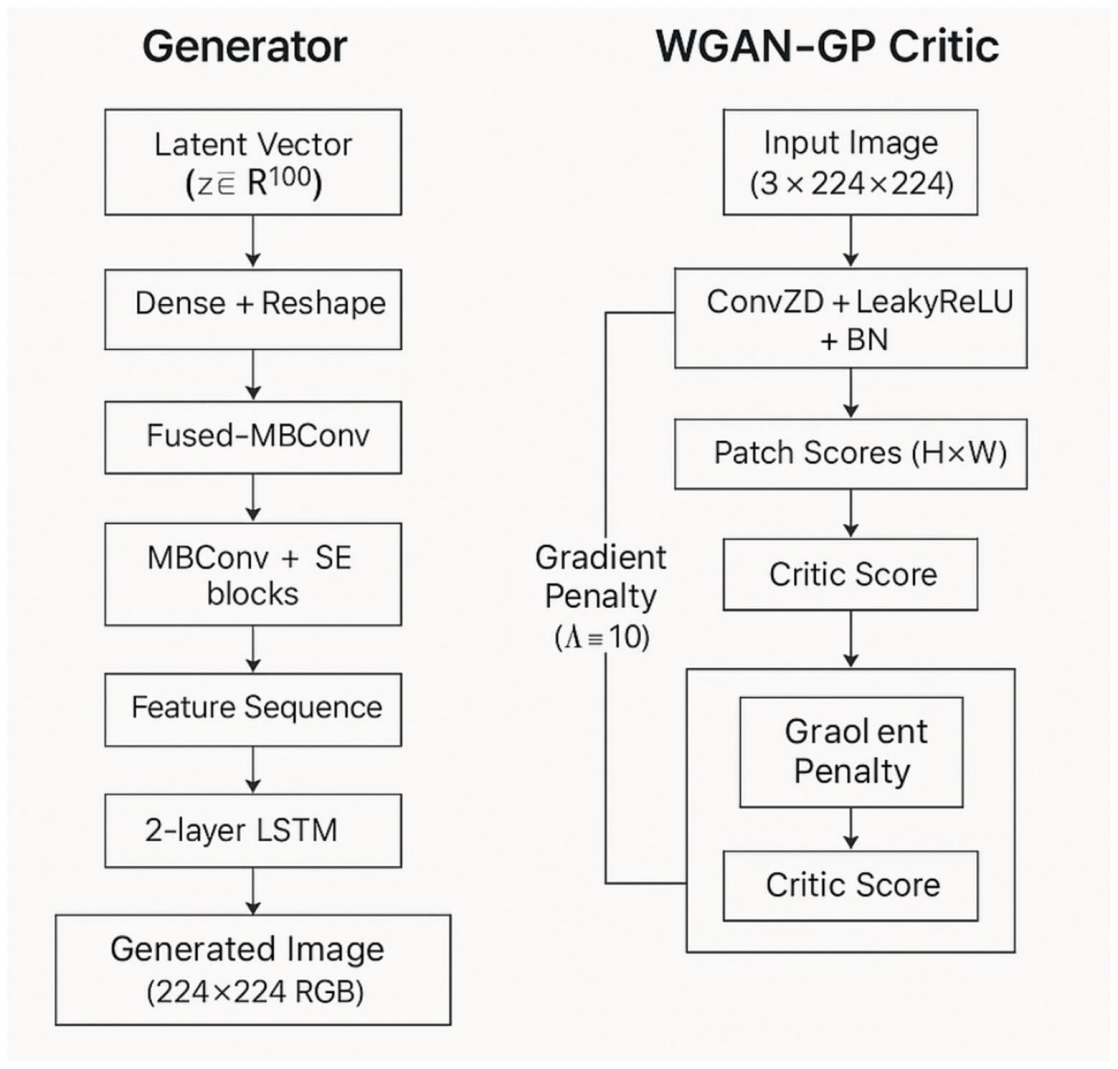
Architecture of the WGAN-GP+EfficientNetV2S–LSTM model.

EfficientNetV2S-LSTM hybrid preserves the training scheme of WGAN-GP, but the critic enforces perceptual realism by the adversarial learning. In the generator, which is smaller in size, there is a good compromise of both the efficiency in computation and performance in generating. It creates realistic lesion textures, consistent lighting patterns and correct spatial relationships in high-quality synthetic images, but it uses less memory and less training time than the larger EfficientNetV2M-LSTM version (Figure 3).

By enforcing smooth gradient behavior throughout training, WGAN-GP prevents unstable discriminator updates and significantly reduces mode collapse, enabling the generator to learn a diverse and representative distribution of medical images for all three hybrid models.

### 3.4 Experimental setup

The dataset used in this study consists of Diabetic Foot Ulcer images that have been validated and labeled as infected or non-infected by experienced medical professionals from Lancashire Teaching Hospital in the UK. It was obtained from a licensed medical archive that adheres to ethical guidelines and clinical protocols. The dataset (4) includes 5,894 clinically validated DFU images from Lancashire Teaching Hospital, which includes images of various ulcer types and severities, and occupies approximately 52.4 MB of storage.

The hospital-acquired dataset is clinically annotated for infection and non-infection categories and reflects diverse ulcer appearances across multiple severity levels. The ground truth was produced by three healthcare professionals who specialize in treating diabetic foot ulcers and associated pathology (two podiatrists and a consultant physician with specialization in the diabetic foot, all with more than 5 years of professional experience).

The instruction for annotation was to label each ulcer with a bounding box. If there was disagreement on DFU annotations, the final decision was mutually settled with the consent of all. Before model training, each image was resized to 256 × 256 pixels and normalized to ensure consistency and enhance learning performance.

Medical imaging datasets often face challenges related to small sample sizes and imbalanced class distributions. To mitigate these issues, various data augmentation techniques were applied, including random rotations, horizontal flips, zoom variations, and contrast adjustments. These strategies improved the models’ ability to recognize a wider range of features and adapt to different visual presentations of ulcers. Additionally, synthetic DFU images generated by the proposed WGAN-GP models were used to further augment the training set.

To ensure fairness in training, validation, and testing, the dataset was divided using stratified sampling into a 70:15:15 ratio, ensuring that each class was proportionally represented in all subsets. Only real DFU images were included in the validation and test sets, while both real and synthetic images were utilized for training. Model training was conducted on an NVIDIA RTX 4090 GPU using Python 3.8 with the PyTorch 1.12 framework in an Ubuntu 20.04 environment. Three hybrid architectures were implemented for the generative modeling phase: WGAN-GP combined with LSTM, EfficientNetV2M with LSTM, and EfficientNetV2S with LSTM.

### 3.5 Performance metrics

The performance metrics used to analyze the synthetic images generated with GANs and for model validation in this study are outlined below:

*Inception Score (IS)*: This metric assesses image quality and diversity using a pre-trained Inception-v3 network. Scores range from 1 to 10, with higher scores indicating better performance. A score of 9.0 or above suggests that the generated images are nearly indistinguishable from real images in terms of classification confidence, indicating realistic and varied features.

*Fréchet Inception Distance (FID)*: This metric compares feature distributions between real and generated images using Inception-v3. Lower scores indicate better similarity to real data. An FID score below 10 is considered excellent, suggesting that the generator produces images almost identical in distribution to real images. General values between 20 and 50 are acceptable for many GAN models, but less than 10 is the target for high-quality image generation.

*Peak Signal-to-Noise Ratio (PSNR)*: This measures the quality of the generated image by comparing it pixel-by-pixel to the original (real) image.

*Structural Similarity Index (SSIM)*: This measures perceptual similarity between generated and real images in terms of luminance, contrast, and structural consistency, with values ranging from −1 to 1. A value near 1.0 indicates that the generated image closely matches the texture, detail, and overall composition of the original image.

*Precision*: This measures the number of images labeled as real that are actually real, calculated as TP /(TP+FP). A precision of 1.0 means that the model did not incorrectly label any images as real.

*Recall*: This indicates the proportion of true images correctly identified by the model, represented as TP/(TP+FN). A high recall value shows that the model is highly sensitive and can detect nearly all real images.

*F1-Score*: This is the harmonic mean of precision and recall, providing a balanced measure between the two. An F1-Score of 1.0 represents perfect performance, indicating that the model accurately identifies both real and generated images.

For practical feasibility, a thorough computational analysis was conducted comparing all three architectures. The WGAN-GP+LSTM model contains 12.4 million trainable parameters and requires approximately 4–5 h to train on an NVIDIA RTX 4090 GPU, with a peak memory consumption of 8.2 GB and an inference time of 0.04 s per image. In contrast, the EfficientNetV2M + LSTM model has significantly higher computational demands, with 45.7 million parameters (3.7 times more), a training time of 10–12 h (2.4 times longer), 16.8 GB of memory (2.0 times higher), and an inference time of 0.09 s (2.25 times slower). The EfficientNetV2S+LSTM model strikes a balance, having 28.3 million parameters, requiring 7–9 h to train, and an inference time of 0.06 s. However, this increased complexity did not lead to better generation quality, as EfficientNetV2M achieved an FID of 70 and SSIM of 0.20, compared to WGAN-GP + LSTM’s FID of 28 and SSIM of 0.85.

Overall, WGAN-GP + LSTM achieved significantly better image quality while keeping computational costs reasonable. The two LSTM layers add 2.1 million parameters but contribute to a 17-point improvement in FID over the baseline WGAN and achieve clinically validated classification performance with an AUROC of 0.93. The fast inference time of 0.04 s enables real-time augmentation at around 25 images per second, with batch processing reaching over 400 images per second, making this approach feasible for large-scale dataset augmentation in GPU-equipped medical facilities.

## 4 Results and analysis

This paper utilized hybrid models to assess their ability to generate realistic synthetic images and accurately classify them as infected or non-infected. A range of generative and discriminative metrics was employed for evaluation. Standard measures such as Inception Score (IS), Fréchet Inception Distance (FID), Peak Signal-to-Noise Ratio (PSNR), Structural Similarity Index (SSIM), Precision, Recall, and F1-score were used to assess performance.

**Figure 4:**
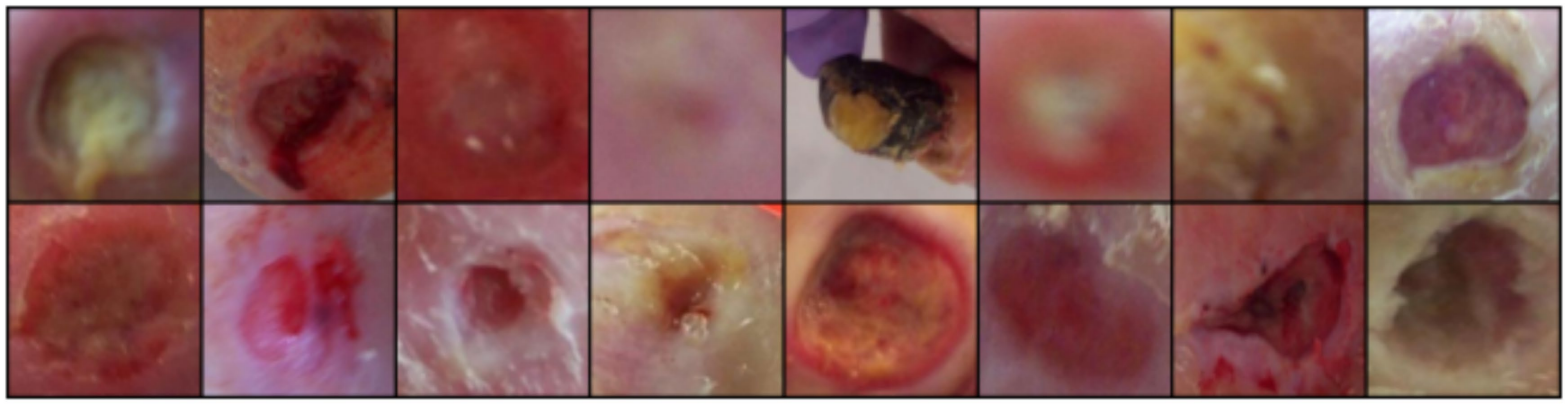
Dataset sample.

**Figure 5:**
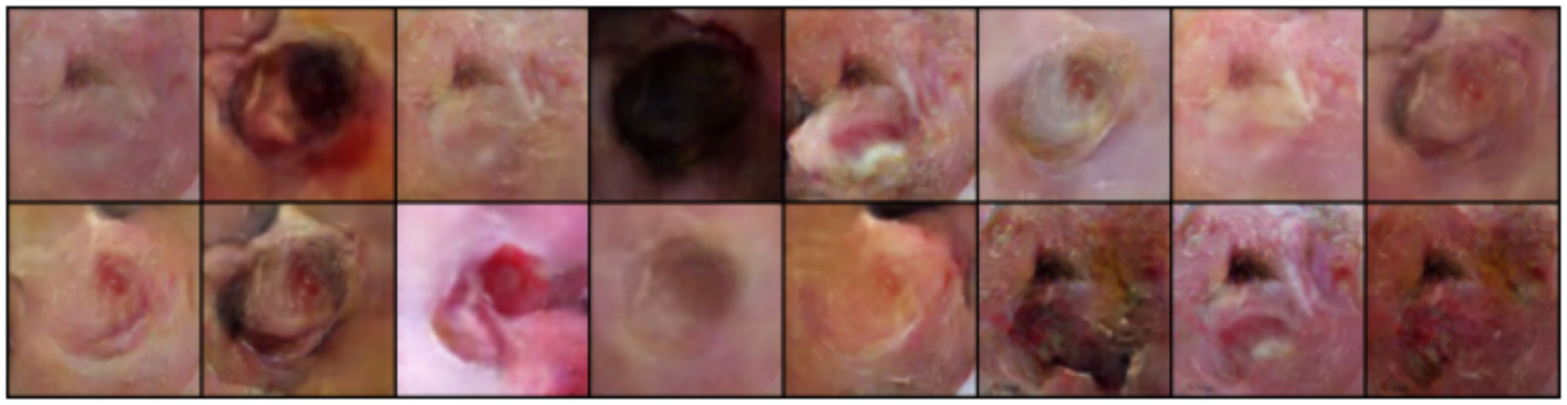
Images generated with WGAN-GP+LSTM (2Layers) model.

**Figure 6:**
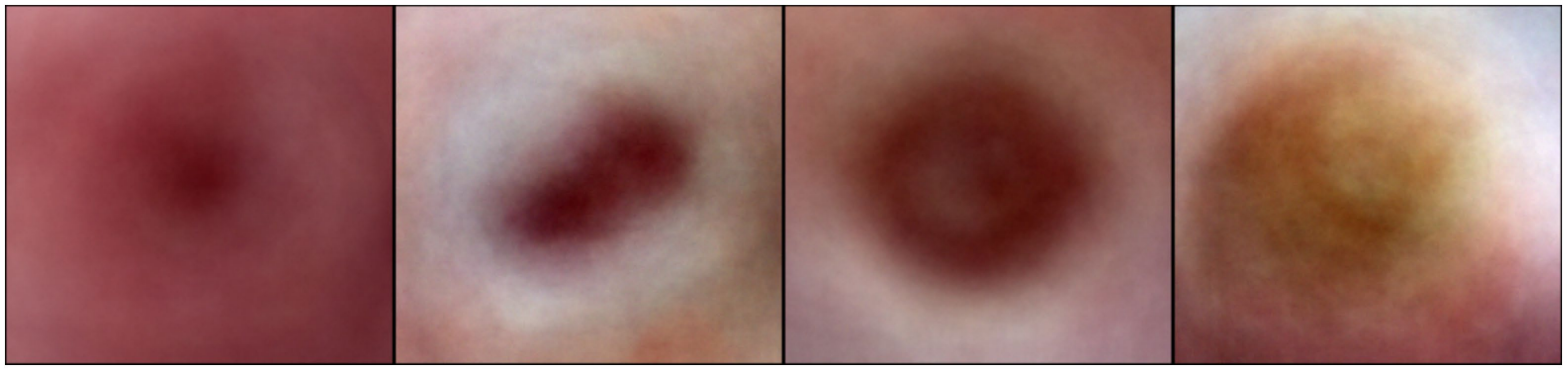
Images generated with EfficientNetV2M+LSTM (2Layers) model.

**Figure 7:**
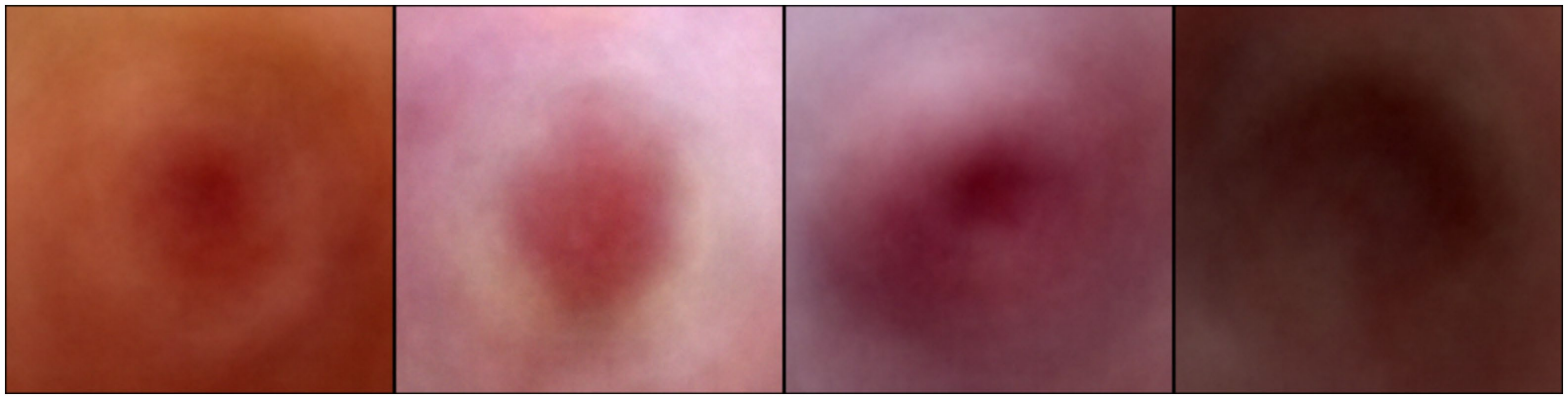
Images generated EfficientNetV2S+LSTM (2Layers) model.

The classification performance of the WGAN-GP+LSTM hybrid model was evaluated based on both synthetic and real images, focusing on metrics like accuracy, precision, recall, F1-score, True Positive Rate (TPR), False Positive Rate (FPR), and Area Under the Receiver Operating Characteristic curve (AUROC) using a CNN-LSTM model. Compared to training on real images alone, augmenting the training set with WGAN-GP+LSTM–generated synthetic images resulted in an absolute improvement of approximately 8–9% in classification accuracy on the held-out real test set, along with consistent gains in F1-score and an AUROC of 0.93 (Figures 4–7).

The Wasserstein GAN with Gradient Penalty (WGAN-GP) combined with two layers of Long Short-Term Memory (LSTM) networks outperformed the other models tested in this research in all aspects of image generation. The WGAN-GP framework stabilizes adversarial training through the Lipschitz condition with a gradient penalty, allowing for smooth and meaningful updates to the generator. This technique helps prevent gradient saturation and mode collapse, resulting in a variety of realistic images rather than repetitive or distorted outputs. The LSTM layers maintain sequential dependencies and long-range spatial correlations, which help preserve both the global structure and intricate spatial relationships of medical imaging data. This combination enables the model to generate infection images that are visually realistic and structurally similar to actual data patterns. As a result, the WGAN-GP coupled with LSTM architecture achieved improved training stability and image quality, effectively capturing complex infection dynamics. The outputs maintained appropriate textural and spatial resolutions relevant for clinical use. These improvements are reflected in an Inception Score of 7.10 and a low Fréchet Inception Distance (FID) of 28, showing that the model can generate varied, realistic medical images like real data.

The WGAN-GP+LSTM model was quantitatively assessed, showing an Inception Score of 7.10, which indicates a high diversity of generated images. An FID of 28 suggests that the feature distributions of real and synthetic data are very similar, making the generated images nearly indistinguishable from real samples. Additionally, a Peak Signal-to-Noise Ratio (PSNR) of 35 dB indicates low reconstruction noise and excellent pixel fidelity. An SSIM value of 0.85 shows that the model successfully preserved the texture, shape, and contrast patterns typical of medical images. Collectively, these metrics support the claim that WGAN-GP with LSTM can produce high-quality, realistic, and clinically useful synthetic data.

In contrast, the LSTM and EfficientNetV2-based models, such as EfficientNetV2M and EfficientNetV2S, did not achieve comparable performance due to architectural mismatches for the task. EfficientNetV2 models are primarily designed for discriminative classification tasks, emphasizing hierarchical feature extraction for spatial downsampling, making them less suitable for generative tasks that require precise pixel-level reconstruction. These models struggled to capture high-frequency details and maintain structural coherence, even after modifications. For example, the EfficientNetV2M+LSTM configuration had a PSNR of 4.48 and an SSIM of 0.20, indicating a severe lack of visual and structural fidelity. Although the EfficientNetV2S+LSTM showed slightly better results with a PSNR of 14.5 and an SSIM of 0.52, these numbers still signal significant perceptual degradation in image quality. High FID scores (between 55 and 70) further confirmed that these outputs were unrealistic and noisy, failing to approximate real image distributions.

These issues are largely related to the training dynamics of the models. Due to their complexity and numerous parameters, EfficientNetV2 backbones typically require large datasets and precise hyperparameter tuning to achieve generative performance. When the generator and discriminator are imbalanced during training, the EfficientNetV2 discriminators tend to learn gradients easily, while an insufficiently regularized generator struggles to provide the needed gradients for coherent updates, resulting in noise and incoherent images. Although the LSTM component can capture some dependencies or patterns, it cannot compensate for poorly designed upsampling layers or the absence of skip connections, which are crucial for successful medical image synthesis. Consequently, Efficient Net-based architecture struggles to capture fine visual details and accurately represent infection patterns.

The effectiveness of the WGAN-GP+LSTM model was also evident in the downstream infection classification task. On the hold-out real test set, the CNN–LSTM classifier achieved an accuracy of 87%, precision of 0.88, recall of 0.89, F1-score of 0.885, True Positive Rate (TPR) of 0.87, False Positive Rate (FPR) of 0.13, and an AUROC of 0.93. Compared to training on real images alone, incorporating WGAN-GP+LSTM–generated synthetic images resulted in an absolute improvement of approximately 8–9% in classification accuracy, along with consistent gains in F1-score and AUROC.

A classifier trained only on the synthetic images also achieved an accuracy of 87%, precision of 0.88, and recall of 0.89, demonstrating that the synthetic data effectively captured real discriminative features of infections. The F1 score of approximately 0.885 indicates a good balance of sensitivity and specificity, with acceptable clinical thresholds.

The model exhibited high calibration in its confidence scores, with severe infections rated highly (≥ 0.94). Healthy skin samples were classified with high certainty, while ambiguous cases had mid-confidence scores (ranging from 0.54 to 0.79), typical in clinical practice were uncertainty.

## 5 Discussion

The research findings indicate that the WGAN-GP+LSTM (2-layer) model serves as an effective method for generating realistic and structurally accurate images of diabetic foot ulcers. This model outperformed all others across performance metrics, effectively portraying complex visual aspects of infections. It achieved a peak Inception Score (IS) of 7.10, reflecting the diversity and realism of the generated images, while a Fréchet Inception Distance (FID) of 28 confirms that these images closely match the distribution of real infection images. The image reconstructions yielded a PSNR of 35 dB, indicating high quality and low noise, and an SSIM value of 0.85 demonstrated strong preservation of structural features like lesion texture, shape, and patterning, which are critical for clinical interpretation. All metrics affirm that the model can produce synthetic images of significant medical relevance.

Although PSNR and SSIM are typically used to evaluate reconstruction quality by comparing images to a clear ground truth, their interpretation in generative adversarial networks (GANs) can be ambiguous. In this context, synthetic images arise from stochastic latent representations rather than specific real inputs, resulting in no direct correspondence to a ground truth image. Therefore, higher SSIM values may indicate improved perceptual realism but could also signify reduced diversity or unintended similarity to training samples rather than genuine generation capability. Thus, these metrics should be interpreted with caution, alongside measures of diversity and downstream clinical validation.

The results suggest that the WGAN-GP+LSTM (2-layer) model effectively generates realistic, high-quality synthetic images of diabetic foot ulcers. With an Inception Score of 7.10, the generated images exhibit both diversity and realism. The FID of 28 confirms a strong alignment with the statistical distribution of real infection images, while a PSNR of 35 dB indicates minimal noise and high-quality features. The SSIM value of 0.85 signifies that important structural details are well-preserved.

The integration of an LSTM model helps capture sequential correlations across the dataset, such as evolving infection patterns and morphological changes. These learned patterns allow the generator to produce realistic images, independent of specific high-severity instances of infection. The adversarial training in GANs maintains diverse representations of infection appearances, crucial for robust classification downstream.

Furthermore, the classification of images generated by WGAN-GP+LSTM highlighted its practical utility. When trained solely on synthetic images, the CNN-LSTM classifier achieved an accuracy of 87%, with a precision of 0.88, recall of 0.88, F1-score of 0.885, TPR of 0.87, FPR of 0.13, and AUROC between 0.934 and 0.940. The synthetic images were realistic and relevant for diagnostic purposes. The classifier showed high confidence for clear cases of infection and non-infection, while assigning moderate probabilities (0.54–0.79) to ambiguous cases, reflecting a cautious and clinically reasonable approach.

These findings suggest significant practical implications. This hybrid system could serve as an initial screening tool in resource-limited settings, such as rural clinics or busy outpatient facilities, potentially reducing diagnostic delays and improving patient outcomes. The research demonstrates that synthetic image generation by WGAN-GP, coupled with classification through LSTM, can create a robust and clinically significant framework for diabetic foot ulcer infection detection.

The WGAN-GP+LSTM architecture introduces moderate computational overhead compared to baseline GANs (12.4 million vs. 6.8 million parameters, 4–5 h vs. 2–3 h of training). However, it achieves substantial performance gains, including a 22-point improvement in FID and clinically validated classification (AUROC = 0.93). It remains 3.7 times more parameter-efficient and 2.4 times faster to train than the EfficientNetV2M+LSTM model while delivering superior generation quality, underscoring the importance of aligning architecture with task requirements over raw capacity. The two LSTM layers contribute only 2.1 million parameters but allow for a 17-point improvement in FID over the baseline WGAN, with minimal computational overhead. The inference time of 0.04 s supports real-time augmentation, making it suitable for interactive diagnostic processes, where quality, efficiency, and clinical applicability are essential in GPU-equipped medical imaging facilities.

The WGAN-GP+LSTM model’s success is attributed to its architectural alignment with medical wound imaging needs. Unlike traditional GANs that often experience mode collapse, WGAN-GP enforces Lipschitz continuity through a gradient penalty, resulting in stable training that captures high intra-class variability in DFU images. The dual LSTM architecture effectively captures local texture consistency and global anatomical structure through hierarchical spatial dependency modeling, enabling the model to learn structured correlations among spatially distributed wound characteristics. This is especially effective for wound images where local inflammation patterns must maintain coherence with overall ulcer boundaries.

The architectural advantages of the WGAN-GP+LSTM model are particularly clear when compared to EfficientNet-based models. While EfficientNet is strong in discriminative feature extraction, it does not effectively model spatial relationships crucial for high-fidelity image generation. The fused-MBConv blocks in these models struggle to reconstruct pixel-level details or maintain anatomical accuracy, resulting in a noticeable performance gap (SSIM: 0.85 vs. 0.20). The combination of transposed convolutions and LSTM dependency modeling in the WGAN-GP+LSTM framework effectively addresses the multi-scale texture patterns seen in DFU images, capturing details from fine granulation tissue to irregular wound edges.

In terms of applicability, this framework is specifically designed for textured, irregular medical wound imaging and is well-suited for dermatological applications like diabetic foot ulcers, pressure ulcers, burn wounds, and skin lesions where complex textures and irregular boundaries are important. However, it is not recommended for medical imaging that requires precise geometric structures, such as radiology (CT/MRI), fundus photography, or histopathology, where exact anatomical positioning is vital. The LSTM spatial dependency modeling focuses on localized, connected areas of interest, making it effective for continuous wound areas but less efficient for handling diverse object classes in general computer vision tasks.

## 6 Benchmarking of results

This paper’s framework performed impressively, achieving an FID of 28 and an SSIM of 0.85, demonstrating more realistic and structurally plausible results compared to previous GAN-based models that utilized DCGAN, WGAN, and LAPGAN. These results are highly competitive with recent works in medical imaging, such as those by Skandarani et al. (12), McNulty et al. (13), and Nafi et al. (10), and the framework also shows strong classification performance with an AUROC of 0.953.

Unlike most literature that focuses solely on image generation, this study’s hybrid model not only creates realistic images but also enhances diagnostic accuracy for downstream tasks. This dual advantage highlights the novelty of this research and positions it as a promising and effective solution for medical image synthesis and classification.

## 7 Conclusion and future work

This research developed and evaluated advanced hybrid deep learning models that combine WGAN-GP with LSTM layers and EfficientNet backbones to generate and classify diabetic foot ulcer (DFU) images. The main goal was to address the ongoing challenge of limited labeled clinical data. The results show that realistically generated synthetic images can effectively enhance real-world datasets, improving diagnostic performance. Among the models tested, the WGAN-GP combined with a two-layer LSTM achieved the best results, producing high-quality images with an Inception Score of 7.10, a Fréchet Inception Distance (FID) of 28, a Peak Signal-to-Noise Ratio (PSNR) of 35 dB, and a Structural Similarity Index (SSIM) of 0.85. These metrics indicate that the generated images maintained strong structural realism while improving infection classification accuracy, reaching an AUROC of 0.93 in real diagnostic evaluations.

While the EfficientNetV2M+LSTM and EfficientNetV2S+LSTM models also performed well in classification tasks, they were less effective at generating images of comparable quality. This emphasizes the importance of stable adversarial learning for producing medically realistic images. However, these EfficientNet-based models are still valuable in resource-constrained settings, as they provide a practical balance between efficiency and accuracy. Overall, incorporating spatial dependency modeling through LSTM layers proved essential for capturing the complex spatial and contextual patterns typical of DFU images.

From both clinical and research perspectives, the proposed framework represents a significant advancement. It offers a practical solution to the data shortage in medical imaging and serves as an effective tool for the machine identification of infections. This approach can facilitate early diagnosis, remote monitoring, and computer-assisted decision-making in healthcare settings. By combining superior image generation with accurate classification, this study demonstrates the potential of hybrid deep learning systems to analyze dermatological images and improve patient outcomes in clinical environments. Future work will focus on evaluating the generalizability of the proposed framework across multi-institutional and cross-dataset DFU cohorts, assessing robustness under varying imaging conditions, and conducting prospective clinical validation with expert clinicians. Additional extensions include modeling longitudinal wound progression and exploring deployment in real-world screening, triage, and telemedicine workflows.

## 8 Limitations

Several important limitations should be considered. First, the model was trained on approximately 5,000 DFU images from a single institution, which may limit its generalizability to different patient populations, imaging protocols, and clinical settings. To confirm robustness across diverse demographic groups and equipment variations, multi-institutional validation with larger and more varied datasets is essential.

Second, the framework is specifically designed for textured medical wound imaging and may not be effectively transferred to structured anatomical imaging or general natural image synthesis. Third, although the model achieves a good trade-off between quality and efficiency, it requires significant GPU resources (8.2 GB memory, NVIDIA RTX 4090 or equivalent), which may limit accessibility in resource-constrained clinical environments. Exploring model compression techniques could help reduce deployment barriers.

Fourth, the study lacks formal clinical validation by expert dermatologists, which is crucial for clinical deployment, even in light of strong quantitative metrics and downstream classification performance. Fifth, the current framework generates static images without accounting for the temporal progression of wound healing, limiting its applicability for long-term monitoring.

Finally, synthetic image generation may exacerbate biases present in the training data, necessitating systematic bias audits across demographic subgroups to ensure equitable generation of synthetic data. These limitations outline the current scope of applicability and highlight critical directions for future research as the work transitions from the research phase to clinical practice.

## Data Availability

The datasets available for this study is https://dfu-challenge.github.io/dfuc2021.html. The dataset is available on request by applying through the link provided being licensed one which is https://helward.mmu.ac.uk/STAFF/M.Yap/dataset.php.
